# The intervention strategies and service model for pharmacist-led diabetes management: a scoping review

**DOI:** 10.1186/s12913-022-08977-1

**Published:** 2023-01-18

**Authors:** Fahmi Hassan, Ernieda Hatah, Adliah Mhd Ali, Chong Wei Wen

**Affiliations:** 1 Faculty of Pharmacy, Universiti Kebangsaan Malaysia, Jalan Raja Muda Abdul Aziz, 50300 Kuala Lumpur, Malaysia; 2 Pharmacy Services Program, Ministry of Health Malaysia, Lot 36 Jalan Universiti, 46350 Petaling Jaya, Selangor Malaysia

**Keywords:** Diabetes, Pharmacist, Non-pharmacological interventions, Patient management

## Abstract

**Background:**

There is increasing intervention activities provided during pharmacist-led diabetes management. Nevertheless, there is an unclear definition of the activities involved during the intervention. Thus, this study aimed to describe the type of intervention strategies and service model provided during pharmacist-led type 2 diabetes management and service outcomes.

**Methods:**

This study utilized the scoping review methodology of the Joanna Briggs Institute Reviewers’ Manual 2015. Articles on pharmacist-led diabetes management focusing on the service content, delivery methods, settings, frequency of appointments, collaborative work with other healthcare providers, and reported outcomes were searched and identified from four electronic databases: Ovid Medline, PubMed, Scopus, and Web of Science from 1990 to October 2020. Relevant medical subject headings and keywords, such as “diabetes,” “medication adherence,” “blood glucose,” “HbA1c,” and “pharmacist,” were used to identify published articles.

**Results:**

The systematic search retrieved 4,370 articles, of which 61 articles met the inclusion criteria. The types of intervention strategies and delivery methods were identified from the studies based on the description of activities reported in the articles and were tabulated in a summary table.

**Conclusion:**

There were variations in the descriptions of intervention strategies, which could be classified into diabetes education, medication review, drug consultation/counseling, clinical intervention, lifestyle adjustment, self-care, peer support, and behavioral intervention. In addition, most studies used a combination of two or more intervention strategy categories when providing services, with no specific pattern between the service model and patient outcomes.

**Supplementary Information:**

The online version contains supplementary material available at 10.1186/s12913-022-08977-1.

## Background

The prevalence of diabetes is increasing worldwide [1]. The prevalence of diabetes in the developed and developing countries is expected to increase by 42% and 170%, respectively, by 2030 [2]. This is particularly a concern in developing countries, as they are commonly affected by rapid population growth, an aging community, unhealthy local diet, urbanization, obesity, unhealthy lifestyle, and poor access to quality health care [3]. As these problems arise, the cost of managing the illness increases. The economic burden of diabetes has necessitated the development of effective interventions that simplify early diagnosis, promote effective care, and enhance primary prevention [4].

The management of diabetes includes maintaining a healthy lifestyle, such as meal planning, physical activity, and medication adherence. Since healthcare providers are not always present, developing self-management skills is critical for diabetes management. Patient education programs have been implemented to educate patients on their active roles in disease management. These programs were reported to result in a better understanding of patients’ perspectives and attitudes toward health, as well as their compliance with drug decisions, risk factors, and overall quality of life [5]. Although pharmacotherapy is an effective treatment modality to achieve optimal glycemic control and prevent the development of diabetes complications, its efficacy is often limited by poor medication adherence among patients with diabetes. Approximately 43.4% of diabetic patients in low- and middle-income countries do not adhere to their pharmacotherapy treatments [6].

An increasing number and types of intervention strategies are being developed to complement pharmacotherapy in diabetes management. Intervention strategies that aim to promote better disease control include patient-mediated strategies through interactions with patients or via the information provided by or to patients [7]. The types of intervention strategies reported in previous studies include counseling, psychological and social interventions, patient empowerment, patient-centered training, explanation of possible adverse events, nutritional therapy, physical activity, and health coaching [8]. Intervention strategies are introduced based on the capacity and needs at the local level and are provided in combination or as single strategies. In addition, patient-centered services facilitated by multiple healthcare professionals, including pharmacists, have shown to enhance outcomes [9–11].

Pharmacists who are knowledgeable in pharmacotherapy are well-trained in identifying patients’ pharmaceutical care issues, such as adverse drug reactions and non-adherence. In addition, pharmacists who are working in outpatient and ambulatory care who are more accessible to the community, are well-positioned to educate, monitor, and support medication adherence and self-care of diabetic patients, which may contribute to the achievement of therapeutic success in diabetes management. An example of a pharmacist-led diabetes management service includes a review of medicines that aim to improve patients’ understanding of the disease and increase their adherence to treatment. Several systematic evaluations have been undertaken throughout the years to investigate the impact of pharmacist-led diabetic care [12–14]. Nonetheless, the studies’ primary focus was on the treatments’ impacts and results, with just a brief mention of the particular tactics and service models delivered. Exploring the specifics of the intervention’s activities and strategies may provide insight into similarities and differences that may or may not have an impact on patient outcomes.

A systematic review by Presley et al. (2018) on interventions to improve medication adherence among adults demonstrated the role of pharmacists in improving diabetes control [8]. In their study, the intervention by pharmacists enhanced diabetes outcomes (standardized mean difference, -0.68; 95% confidence interval, -0.79, -0.58; p < 0.001) with subgroup analysis by intervention strategy, and the type of intervention and outcome measures produced similar results. In their study, nevertheless, the intervention strategies were classified as educational, behavioral, or a combination of both, with an unclear definition of the activities involved. Since many different activities were reported to be provided during pharmacist-led diabetes management services, it is worth exploring and classifying intervention strategies based on their specific activities [8]. In addition, the effectiveness of these two broad intervention strategies was inconsistent between studies in which the best interventions to improve nonadherence could not be determined [8]. Thus, this study aimed to provide a review of the type of individualized intervention strategies and service model provided during pharmacist-led type 2 diabetes management, which includes reviewing specific service content, such as information on the activities involved, delivery methods, settings, frequencies of appointments, and collaborative work with other healthcare providers and their outcomes.

## Methods

As the study aimed at providing an overview of the intervention strategies and service model provided during pharmacist-led type 2 diabetes management, scoping review methodology was deemed the most appropriate method to be applied. The current study utilized the scoping review methodology of the Joanna Briggs Institute Reviewers’ Manual 2015 [15]. The manual was one of the latest published on scoping reviews methodology. The step-by-step approach in the manual was well described and provided clear guidance for conducting a scoping review. Articles on pharmacist-led diabetes management focusing on service content, delivery methods, settings, frequencies of appointments, collaborative work with other healthcare providers, and reported outcomes were searched and identified.

### Search criteria

Articles were searched from four electronic databases: Ovid Medline, PubMed, Scopus, and Web of Science from 1990 to October 2020. Relevant Medical Subject Headings (MeSH) and keywords such as “diabetes,” “medication adherence,” “blood glucose,” “HbA1c,” and “pharmacist” were used to identify published articles. The specific search strings used for the search can be found in the supplementary material. To increase the specificity and sensitivity of the identified articles, MeSH terms and keywords were combined using the Boolean operator, AND or OR, where appropriate. The reference lists of the retrieved papers were screened for potentially relevant papers that were missing during the electronic search.

### Study selection

All retrieved articles were imported to Mendeley, a reference management system software, and duplicates were removed. Articles were included if they had been conducted as a randomized controlled trial; intervention conducted by pharmacists aimed at improving medication adherence and/or glycemic control, including diabetic patients aged 18 and above; and conducted in outpatient or ambulatory settings and reported glycemic control or medication adherence level as the outcomes. Multicomponent team-based care and programs were included if the study defined the program as a pharmacist-led program and the involvement of other healthcare providers are only to address specific issues such as prescribing and meal planning. Only studies with randomized controlled trials (RCTs) study design were included as they usually represent optimal study design and regarded as highest quality evidence. RCTs usually minimize bias in their study procedure and combining RCTs with other study designs may increase study’s heterogeneity, hence, making comparison and conclusion difficult to be made. In addition, randomized controlled trials report usually includes a more detailed information on their intervention programs making it possible for relevant and accurate details to be extracted. Articles were excluded if they had been conducted in a well-controlled environment, such as in a university or academic institution that did not reflect a real practice setting; full articles could not be retrieved; and were not published in the English language.

Articles were initially screened by F.H. based on their title and abstract. The exclusion process using titles or abstracts by F.H. occurred only if the reason for exclusion was clear. If there was uncertainty, the article was not excluded, and each member of the research team (F.H., E.H., A.M.A., C.W.W.) reviewed the article. All excluded “full text” articles were independently reviewed by F.H., E.H., A.M.A., and C.W.W. to ensure the validity of the process. Any disagreements regarding whether a study should be included or excluded were resolved through consensus when the majority indicated their agreement over the matter.

### Data extraction

F.H. performed data extraction for all articles, which was checked by E.H. Extracted data included title, year of publication, authors and location of the study, contents of the intervention, delivery approaches, healthcare workers involved, frequency of appointments, follow-up period, and reported outcomes. C.W.W. and A.M.A. reviewed the extracted data in a table form. Discrepancies were discussed and resolved by consensus.

### Data analysis

Data analysis was conducted through a narrative synthesis of the articles by evaluating and comparing the pharmacist-led interventions reported in the articles. The results were summarized according to the type of service content, delivery methods and settings, frequency of appointments, collaborative work with other healthcare providers, and outcomes of glycemic control and medication adherence. The types of service content and delivery methods with their definitions were identified from the studies based on the description of activities reported in the articles and are tabulated in the summary table. The code was initially categorized by F.H. and refined by E.H. The final coding was assessed by all team members, F.H., E.H., A.M.A., and C.W.W., and disagreement was resolved through consensus. The current study reported that the glucose control outcome as significant if at least one of the result of the glucose readings measured by the studies (random blood glucose, fasting blood glucose, HbA1c or post-prandial blood glucose) was reported significant.

## Results

**Fig. 1:**
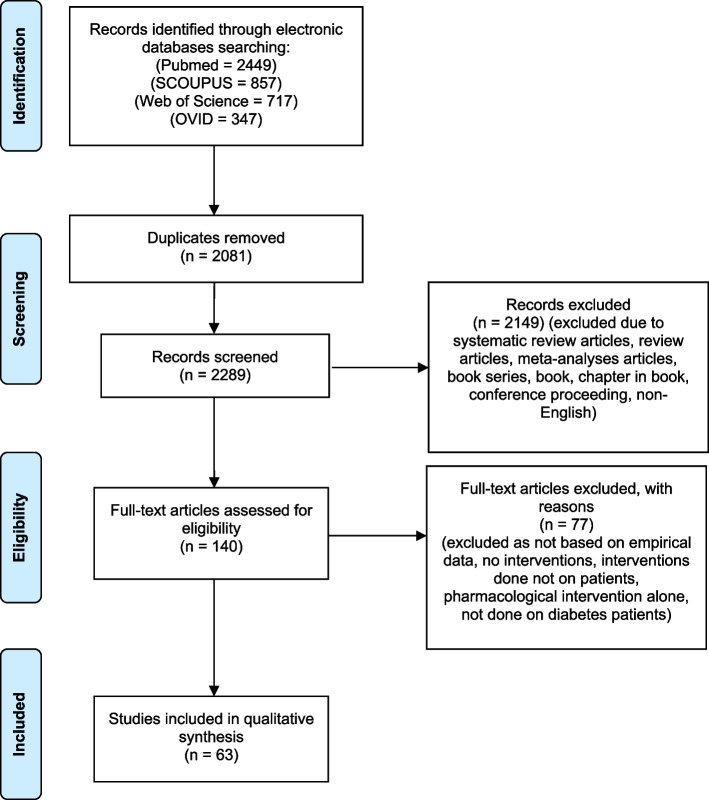
Flow chart of the search result

The systematic search retrieved 4,370 articles located through the electronic database search. After removing duplicates and titles/abstracts that were unrelated to pharmacist-led diabetes management, 140 articles were included in the full-text review. After applying the inclusion and exclusion criteria, 61 articles were included in the analysis. Figure 1 summarizes the PRISMA flow process for the identification, screening, and inclusion of the identified articles.

All included studies were randomized controlled trials. Studies included were from Australia (*n* = 2), South America (*n* = 4), North America (*n* = 16), Europe (*n* = 7), Asia (*n* = 27), and Africa (*n* = 2). The earliest study was published in 1996, and most studies (*n* = 48) were published after 2010. Pharmacist interventions on diabetes management were provided in settings, such as community health centers (*n* = 4), community pharmacies (*n* = 11), outpatient clinics (*n* = 44), and outpatient pharmacies (*n* = 2). Table 1 summarizes the characteristics of the included studies.

**Table 1:** Summary of studies and description of service model and intervention provided

No.	Author	Country	Year	Location	HCP involvement		Method of delivery				Tools/ Aids					
					Pharmacist	Multidisciplinary Tteam	Face to face	Phone calls	Group sessions	Home visits	Diaries	Printed materials	Video	Email	Pill box	Games
1	Castejon et al.	USA	2013	Outpatient Pharmacy	x		x		x				x			
2	Lim et al.	Malaysia	2016	Outpatient Clinic		x	x				x					
3	Ali et al.	UK	2012	Community Pharmacy		x	x				x					
4	Venkatesan et al.	India	2012	Community Pharmacy	x		x					x				
5	Grant et al.	USA	2003	Community Health Center		x		x						x		
6	Ramanath et al.	India	2012	Outpatient Clinic	x		x					x				
7	Mahwi et al.	Iraq	2013	Outpatient Clinic	x		x									
8	Clifford et al.	Australia	2002	Outpatient Clinic		x	x									
9	Phumipamorn et al.	Thailand	2008	Outpatient Clinic		x	x									
10	Al Mazroui et al.	UAE	2009	Outpatient Clinic		x	x				x	x				
11	Farsaei et al.	Iran	2011	Outpatient Clinic	x		x	x			x				x	
12	Mehuys et al.	Belgium	2011	Community Pharmacy	x		x									
13	Jacobs et al.	USA	2012	Outpatient Clinic		x	x									
14	Jarab et al.	Jordan	2012	Outpatient Clinic		x	x	x				x				
15	Odegard et al.	USA	2012	Community Pharmacy	x		x	x								
16	Shah et al.	USA	2013	Outpatient Clinic	x		x									
17	Chung et al.	Malaysia	2014	Outpatient Clinic		x	x	x							x	
18	Jahangard et al.	Iran	2015	Community Pharmacy		x	x	x			x	x				
19	Wishah et al.	Jordan	2015	Outpatient Clinic		x	x	x				x				
20	Xin et al.	China	2015	Outpatient Clinic	x		x	x	x			x				
21	Butt et al.	Malaysia	2016	Outpatient Clinic	x		x									
22	Chen et al.	Taiwan	2016	Outpatient Clinic		x	x	x		x					x	
23	Aguiar et al.	Brazil	2016	Outpatient Clinic		x	x	x			x	x				
24	Chow et al.	Malaysia	2015	Outpatient Clinic	x			x		x	x	x				
25	Renuga et al.	India	2016	Outpatient Pharmacy	x		x					x				
26	Samtia et al.	India	2013	Outpatient Clinic	x		x									
27	Cani et al.	Brazil	2015	Outpatient Clinic		x	x								x	
28	Chan et al.	Hong Kong	2012	Outpatient Clinic		x	x								x	
29	Abuloha et al.	Jordan	2016	Outpatient Clinic		x	x	x			x					
30	Choe et al.	USA	2005	Outpatient Clinic		x	x	x								
31	Clifford et al.	Australia	2005	Outpatient Clinic		x	x	x								
32	Cohen et al.	USA	2011	Outpatient Clinic		x			x		x	x				
33	Doucette et al.	USA	2009	Community Pharmacy		x	x									
34	Fornos et al.	Spain	2006	Community Pharmacy		x	x					x				
35	Ghosh et al.	India	2010	Outpatient Clinic		x	x					x				
36	Jaber et al.	USA	1996	Outpatient Clinic		x	x									
37	Kraemer et al.	USA	2012	Community Health Centre	x		x									
38	Mourao et al.	Brazil	2013	Outpatient Clinic		x	x									
39	Plaster et al.	Brazil	2012	Outpatient Clinic		x	x	x				x				
40	Sriram et al.	India	2011	Outpatient Clinic	x		x	x			x	x				
41	Taveira et al.	USA	2010	Outpatient Clinic		x			x							
42	Jameson et al.	USA	2010	Outpatient Clinic		x	x	x								
43	Odegard et al.	USA	2005	Outpatient Clinic		x	x	x								
44	Kirwin et al.	USA	2010	Outpatient Clinic		x	x									
45	Lyons et al.	UK	2016	Community Pharmacy	x			x				x				
46	Malathy et al.	India	2011	Outpatient Clinic	x		x					x				
47	Rothman et al.	USA	2005	Outpatient Clinic		x	x	x								
48	Sarkadi & Rosenqvist et al.	Sweden	2004	Community Health Center		x			x		x		x			x
49	Scott et al.	USA	2006	Community Health Center		x	x	x	x							
50	Adepu et al.	India	2007	Community Pharmacy	x		x					x				
51	Erku et al.	Ethiopia	2017	Outpatient Clinic		x	x	x								
52	Korcegez et	Cyprus	2017	Outpatient Clinic		x	x					x				
53	Ojieabu et	Nigeria	2017	Outpatient Clinic	x		x	x								
54	Shao et al.	China	2017	Outpatient Clinic	x		x	x								
55	Lauffenburger et al.	USA	2019	Outpatient	x			x							x	
56	Wu et al.	USA	2018	Outpatient Clinic		x			x							
57	Michiels et al.	France	2019	Community Pharmacy	x		x					x				
58	Javaid et al.	Pakistan	2019	Outpatient Clinic		x	x									
59	Withidpanyawong et al.	Thailand	2018	Outpatient Clinic	x				x							
60	Sarayani et al.	Iran	2018	Community Pharmacy	x			x								
61	Ayadurai et al.	Malaysia	2018	Outpatient Clinic		x	x									

No.	Author	Country	Year	Location	Content								Outcome	
					Diabetes education	Medication review	Drug counselling	Clinical intervention	Lifestyle adjustment	Self care	Peer support	Behavioural intervention	Glucose control	Adherence
1	Castejon et al.	USA	2013	Outpatient Pharmacy	x		x			x		x	I > C	
2	Lim et al.	Malaysia	2016	Outpatient Clinic		x		x					I > C	I > C
3	Ali et al.	UK	2012	Community Pharmacy	x	x							I > C	
4	Venkatesan et al.	India	2012	Community Pharmacy	x		x		x	x			NS	
5	Grant et al.	USA	2003	Community Health Center			x						NS	NS
6	Ramanath et al.	India	2012	Outpatient Clinic	x								I > C	I > C
7	Mahwi et al.	Iraq	2013	Outpatient Clinic		x	x						I > C	I > C
8	Clifford et al.	Australia	2002	Outpatient Clinic	x								NS	
9	Phumipamorn et al.	Thailand	2008	Outpatient Clinic	x	x			x				I > C	I > C
10	Al Mazroui et al.	UAE	2009	Outpatient Clinic	x				x	x			I > C	
11	Farsaei et al.	Iran	2011	Outpatient Clinic	x	x	x			x			I > C	
12	Mehuys et al.	Belgium	2011	Community Pharmacy	x		x		x				I > C	
13	Jacobs et al.	USA	2012	Outpatient Clinic	x	x			x	x			I > C	
14	Jarab et al.	Jordan	2012	Outpatient Clinic	x	x			x			x	I > C	
15	Odegard et al.	USA	2012	Community Pharmacy			x			x				I > C
16	Shah et al.	USA	2013	Outpatient Clinic	x					x			I > C	I > C
17	Chung et al.	Malaysia	2014	Outpatient Clinic	x	x				x			I > C	I > C
18	Jahangard et al.	Iran	2015	Community Pharmacy	x				x	x			NS	I > C
19	Wishah et al.	Jordan	2015	Outpatient Clinic	x				x	x			I > C	
20	Xin et al.	China	2015	Outpatient Clinic	x								I > C	I > C
21	Butt et al.	Malaysia	2016	Outpatient Clinic	x		x		x	x			I > C	I > C
22	Chen et al.	Taiwan	2016	Outpatient Clinic	x	x							I > C	
23	Aguiar et al.	Brazil	2016	Outpatient Clinic	x				x	x			I > C	I > C
24	Chow et al.	Malaysia	2015	Outpatient Clinic	x				x	x			I > C	I > C
25	Renuga et al.	India	2016	Outpatient Pharmacy	x								I > C	I > C
26	Samtia et al.	India	2013	Outpatient Clinic	x				x	x			NS	I > C
27	Cani et al.	Brazil	2015	Outpatient Clinic	x	x		x	x	x			NS	I > C
28	Chan et al.	Hong Kong	2012	Outpatient Clinic	x	x							I > C	I > C
29	Abuloha et al.	Jordan	2016	Outpatient Clinic	x		x		x	x			I > C	
30	Choe et al.	USA	2005	Outpatient Clinic	x	x		x		x			I > C	
31	Clifford et al.	Australia	2005	Outpatient Clinic		x			x	x			I > C	
32	Cohen et al.	USA	2011	Outpatient Clinic	x	x				x	x		NS	
33	Doucette et al.	USA	2009	Community Pharmacy		x		x		x			NS	
34	Fornos et al.	Spain	2006	Community Pharmacy	x	x							I > C	
35	Ghosh et al.	India	2010	Outpatient Clinic	x	x			x				I > C	
36	Jaber et al.	USA	1996	Outpatient Clinic	x	x			x	x			I > C	
37	Kraemer et al.	USA	2012	Community Health Centre		x			x	x			NS	
38	Mourao et al.	Brazil	2013	Outpatient Clinic	x	x			x	x			I > C	
39	Plaster et al.	Brazil	2012	Outpatient Clinic		x			x				I > C	
40	Sriram et al.	India	2011	Outpatient Clinic		x			x				I > C	
41	Taveira et al.	USA	2010	Outpatient Clinic	x	x		x	x	x		x	I > C	
42	Jameson et al.	USA	2010	Outpatient Clinic	x	x		x		x			NS	
43	Odegard et al.	USA	2005	Outpatient Clinic		x							NS	
44	Kirwin et al.	USA	2010	Outpatient Clinic		x		x					NS	
45	Lyons et al.	UK	2016	Community Pharmacy			x						NS	I > C
46	Malathy et al.	India	2011	Outpatient Clinic	x				x				NS	
47	Rothman et al.	USA	2005	Outpatient Clinic	x	x		x				x	NS	
48	Sarkadi & Rosenqvist et al.	Sweden	2004	Community Health Center	x	x				x			I > C	
49	Scott et al.	USA	2006	Community Health Center	x	x		x		x			I > C	
50	Adepu et al.	India	2007	Community Pharmacy	x				x	x			NS	
51	Erku et al.	Ethiopia	2017	Outpatient Clinic	x	x			x				I > C	
52	Korcegez et	Cyprus	2017	Outpatient Clinic	x	x		x	x	x			I > C	I > C
53	Ojieabu et	Nigeria	2017	Outpatient Clinic	x				x				I > C	I > C
54	Shao et al.	China	2017	Outpatient Clinic	x				x	x			I > C	I > C
55	Lauffenburger et al.	USA	2019	Outpatient					x			x	NS	NS
56	Wu et al.	USA	2018	Outpatient Clinic	x		x			x		x	NS	
57	Michiels et al.	France	2019	Community Pharmacy	x		x						I > C	NS
58	Javaid et al.	Pakistan	2019	Outpatient Clinic	x	x	x		x				I > C	
59	Withidpanyawong et al.	Thailand	2018	Outpatient Clinic	x		x						I > C	I > C
60	Sarayani et al.	Iran	2018	Community Pharmacy	x					x			NS	I > C
61	Ayadurai et al.	Malaysia	2018	Outpatient Clinic	x	x			x				I > C	

Most interventions were conducted by multidisciplinary teams of healthcare providers, including pharmacists, doctors, nurses, dietitians, and diabetes educators (*n* = 37). Only 24 of the included studies contained interventions performed solely by pharmacists. In such settings, the interventions conducted by pharmacists, which are rarely clinical interventions, include diabetes education, medication review, drug counselling, self-care recommendations, and lifestyle adjustments.

The method of service delivery included face-to-face sessions with individual patients, which was the most common method used (*n* = 51), scheduled telephone calls (*n* = 27), group sessions (*n* = 8), and visits to patients’ homes (*n* = 2). Fifteen studies used face-to-face meetings as the single method to deliver interventions [16–30]. Another four studies utilized single delivery group sessions [31–33] and phone calls [34] to deliver interventions. Three studies used a combination of three intervention delivery methods [35–37]. The most common combination of delivery methods was face to face meeting and phone calls (*n* = 22) [35, 37–56]. During the interventions, several delivery aids were used, such as videos (*n* = 2), printed materials consisting of a summary of important information for patients (*n* = 20), email reminders (*n* = 1), patient diaries (*n* = 11), and pillboxes (*n* = 6).

Eight intervention strategy categories were identified in the included studies. Table 2 provides a detailed description of the categories and potential activities involved. Intervention strategies were categorized into diabetes education, medication review, drug consultation/counseling, clinical intervention, lifestyle adjustment, self-care, peer support, and behavioral intervention. The most popular strategy for this was diabetes education. The educational components of these interventions primarily aimed to increase the patients’ general understanding of their condition by discussing the expected degree of diabetic control, risk of complications, and ways to minimize these risks [57]. Patients were also informed about the types of medications used to treat their disease [58].

**Table 2:** Description of intervention categorization and potential activities involved

Intervention categories	Description of intervention	Example of activities involved	References
Diabetes education	Provide the patient with adequate knowledge about diabetes and skills they need to manage their clinical condition and lifestyle.	Educational videos, pamphlets, educational websites, power point presentations, face-to-face/group teaching sessions.	[14–20, 22, 24, 26–34, 38–44, 46–51, 53, 56, 57, 60, 61, 63–74]
Medication review	Pharmacists addressed issues pertaining to medication optimization and adherence, hence enhancing the therapeutic efficacy of drugs administered to patients.	Review of patients’ medications, arrangements of drug taking schedules, discussion and evaluation of medication regimens, dose up-titrations per pre-established protocols without prescriber’s involvement.	[10, 13, 15, 17, 21–28, 33–41, 44, 46, 48, 52, 53, 60, 64, 68, 70, 72–74]
Drug counseling	Aids patients in the comprehension of medications and is emphasises the importance of drug adherence.	Evaluation of patients’ medication adherence, pill counts, medication diaries, pill boxes, pill reminder apps.	[13, 16, 19, 26, 29, 30, 37, 49, 53, 58, 59, 63, 69, 71]
Clinical review	A collaborative intervention with the prescriber on drug related problems requiring clinical interventions such as regimen changes or dosage adjustment.	Evaluation and adjustment of patients’ medication with the involvement of the prescribers.	[10, 21, 25, 28, 34, 39, 40, 44, 72, 73]
Lifestyle adjustment	Focused primarily on healthy eating and encouraging patients to lead a more active lifestyle.	Exercise prescription, specific diet recommendation.	[15–17, 19, 20, 22–24, 26–28, 35, 36, 41–43, 46, 47, 49–52, 61, 62, 64–67, 69, 72, 73]
Self-care	Approaches to manage and prevent diabetes complications through self-blood glucose monitoring and foot care.	Glucose diaries, glucose monitoring device program, proper foot care program.	[17–24, 28, 29, 31, 34, 35, 37, 39, 43, 44, 47–51, 53, 65–73]
Peer support	Promotes communications and sharing of knowledge and experience between patients that have poor control of diabetes with patients that already have better experience in managing their disease.	Participation of family members, friends, and other sources of social support in the intervention program.	[70]
Behavioural intervention	Incorporate behaviour-change techniques such as goal-setting, cognitive behavioural therapy, and problem-solving.	Predetermined action items, action planning, motivational interviewing.	[28, 29, 40, 46, 62, 71]

Only seven studies utilized a single intervention strategy, which included diabetes education [17, 35, 59, 60], medication review [37], and drug consultation/counseling [61, 62]. Most studies incorporated two or more intervention strategy categories. In particular, 17 combined two strategies [16, 28, 33, 34, 36, 39, 41, 45, 48, 55, 63–66], 18 combined three [18, 19, 23, 24, 26, 30, 38, 44, 46, 50, 51, 53, 54, 67–71], 16 combined four [20, 22, 25, 27, 29, 32, 37, 42, 43, 47, 49, 52, 56, 72–74], 2 combined five [75, 76], and 1 combined six [31]. The most commonly utilized intervention strategy was diabetes education (n = 49), whereas the least utilized service content category was peer support (*n* = 1).

The follow-up periods of the pharmacists’ interventions differed in each study and ranged between 1.5 [67] and 24 months [42]. One study followed up patient for 2 months [59], nine for 3 months [16, 52, 53, 56, 60, 61, 64, 70, 74], five for 4 months [21, 25, 31, 45, 68], three for 5 months [23, 50, 53], fourteen for 6 months [22, 27, 30, 36, 39, 40, 44, 46, 49, 62, 73, 75], three for 8 months [18, 55, 72], four for 9 months [29, 33, 37, 41], eighteen for 12 months [20, 24, 26, 28, 35, 38, 43, 47, 48, 51, 54, 63, 65, 66, 69, 71, 76, 77], one for 13 months [32] and another one for 16 months [10]. The most common follow-up period for the interventions was 12 months (n = 19, 31.1%), and the mean intervention duration was 7.8 months.

The frequency of follow-up varied from a minimum of a single follow-up [21, 28, 61, 69] to 24 follow-ups [42]. Five studies set a frequency of two follow-ups [44, 48, 59, 62, 68], twelve set three follow-ups [16, 20, 22, 29, 49, 52, 60, 64, 65, 70, 72, 74], five set four follow-ups [17, 18, 24, 31, 45], two set five follow-ups [53, 76], eleven set six follow-ups [19, 26, 27, 30, 33, 36, 39, 54, 67, 75, 77], four set eight follow-ups [10, 32, 37, 38], another four set nine follow-ups [40, 46, 66, 73], one set ten follow-ups [25], and six set twelve follow-ups [35, 43, 51, 56, 63, 71]. Five of the studies did not describe the number of follow-ups carried out in detail [23, 41, 47, 50, 55]. The mean number of follow-ups that the patients received was six. The most common number of follow-ups reported by the studies was three (*n* = 13, 21.43%).

Most studies (*n* = 36) reported glucose control as the outcome, 24 reported glucose control and medication adherence as the outcomes, and one study reported medication adherence as the outcome. Most studies (*n* = 41) also showed significant improvement in glucose control, which was measured by glycosylated hemoglobin, fasting or random blood glucose levels, or a combination of these. Meanwhile, 22 of the 25 studies reported significant improvement in medication adherence measured using the eight-item Morisky Scale, Malaysian Medication Adherence Scale, pill-count, self-reported adherence scale, dispensing history, diagnostic adherence to medication scale, or Morisky Green Levine Medication Adherence Scale.

## Discussion

The current scoping review aimed to evaluate the type of interventions and service model provided during the provision of pharmacist-led type 2 diabetes management which previously had unclear classification with no detail on the activities involved. This includes a review of the type of service content, delivery methods, settings, frequencies of appointments, collaborative work with other healthcare providers, and reported outcomes. Pharmacist-led diabetes interventions were provided in six continents, with most studies (*n* = 37) conducted in Asia. This was not surprising since the highest prevalence of diabetes is found in Asian countries [1]. Thirty of the studies were conducted in high-income countries, one in low-income country, 13 in lower-middle-income countries, and 17 in upper-middle-income countries. There is a lack of interest in the topic in low-income countries even though the prevalence of non-adherence towards treatment is high and the promotion of effective treatment plan would help reduce the burden of diabetes management in such countries [4, 6]. Most services (*n* = 37) were provided by a multidisciplinary health care team, and only a few (*n* = 24) were conducted solely by pharmacists. Nevertheless, in these studies, most showed that pharmacists also worked directly with a physician on patient issues, for example, if the patient required approval for prescription adjustment and specific diet plan such as fasting. A meta-analysis study on the multidisciplinary team approach to coordinated pharmaceutical care found that such collaborations reduced the likelihood of patients’ hospitalization and increased their quality of life [78].

Face-to-face sessions were the most common method for pharmacist-led diabetes management reported in the included studies. This traditional method of service delivery is well established and generally well accepted by patients. Most studies reported good patient retention throughout the study period. However, unlike in a trial environment, patients in a real-world setting may not be able to complete proper follow-ups with frequent face-to-face appointments. In clinical trials, patients are routinely reminded to attend their subsequent appointments and are often rewarded with tokens for their participation in the trials. Therefore, they might have different motivations for retaining themselves in the program compared to actual patients outside clinical trial settings. For example, in a diabetes prevention program in England involving 100,000 patients, only 22% of the participants completed the program [79]. Therefore, it is important to review an intervention program beyond the “controlled” environment and ensure its convenience for the patient. Providing more interventions through phone calls and video conferences should be explored in the future, as they are generally equally effective as face-to-face sessions [80]. Home visits may suit patients with logistics issues who require frequent clinic visits. Meanwhile, group sessions may be useful for behavioral interventions that include peer support and mentoring activities [81].

Eight categories of pharmacist-led service contents were identified from the reported studies, which included diabetes education, medication review, drug consultation/counseling, clinical intervention, lifestyle adjustment, self-care, peer support, and behavioral intervention. The majority of the studies combined two or more service content categories for intervention. Although the best combination of interventions for diabetes management could not be determined due to an inconsistency in the services provided across the studies, combining several types of intervention content was found to be more effective than a single intervention [8, 82]. Studies have shown that a combination of services improves patients’ medication adherence and glucose control. In the current study, diabetes education prevailed as the most common intervention in pharmacist-led diabetes services, with the aim of providing patients with the knowledge and skills needed to manage their clinical condition and lifestyle. Previous systematic reviews also found that diabetes education was most effective in improving diabetes control and enhancing medication adherence [8, 57, 58, 82]. During diabetes education, printed or digital materials and training or group discussions can also be considered, as they were also reported to be equally effective [8].

In the current study, medication review was the second intervention widely used during pharmacist-led diabetes management. During medication review, pharmacists optimize patients’ medications and ensure their adherence [83]. Medication review is one of the most effective tools for improving patient clinical outcomes and safety by resolving treatment complexities. Several systematic reviews have reported that medication reviews in the care of diabetic patients have improved clinical results and provided favorable economic outcomes that are not only beneficial to the self-paying patients, but also to the healthcare system [84, 85]. Other service interventions include self-care content, such as self-blood glucose monitoring and foot care; lifestyle adjustments, such as eating habits and physical exercise; drug consultation or counseling that focuses on effective use of medications; clinical intervention that includes a change in regimen or dosing adjustment that was carried out after agreement with the prescriber; and behavior-change content, such as goal-setting, cognitive behavioral therapy, and problem solving. The majority of the studies employed a variety of terminology to name their intervention techniques. This makes determining the types of specific intervention delivered challenging. A list of standardized terminologies and their meanings would be extremely valuable for practice harmonization and guaranteeing that future intervention program outcomes may be compared in a more methodical and meaningful manner.

The current study found that the duration and frequency of pharmacist-led diabetic interventions varied between the studies. A previous review found that studies with longer follow-up periods were associated with better outcomes [8]. Nevertheless, most included studies had good patient retention rates throughout the study period, which could differ in actual practice; the reason for the difference could be due to the “controlled environment” in the trial setting, in which patients were closely followed up. The same study also reported that pharmacist interventions significantly improved most of the outcome measures within three follow-up visits [8]. Hence, the delivery of content should be based on the patient’s immediate needs and should not be too structured in the view that patients may not return for their next appointment. In addition, no consistent pattern was found between intervention categories and patient outcomes. This would be difficult to identify because the majority of the included studies reported significant improvements in glucose control and medication adherence.

This study has a few limitations. Firstly, since the intended purpose of a this scoping review is to gather information on type of intervention strategies provided during pharmacist-led diabetes management, as opposed to recommending a clinical practice, quality assessment is not undertaken hence, making it impossible for any implications for practice or policy to be graded. The components of the interventions and their categorization were based on the information provided in the manuscript. Some interventions were not sufficiently explained, which may have caused limitations in the intervention categorization. However, we independently checked the assigned categories and ensured that the intervention components were identified appropriately. In addition, most included studies reported significant findings on glucose control and improvement in medication adherence, making it difficult to evaluate the effectiveness of individual intervention categories. The risk of bias assessment was not done to evaluate the study quality, as this study aimed only to provide an overview of intervention categories according to the activities described in the study.

## Conclusion

Variations in intervention strategies were found in the reported studies, with the most used being diabetes education and medication review. Most studies used a combination of two or more intervention strategy categories when providing services, with no specific pattern found between the service model and patient outcomes. A standardization of terminologies used for future pharmacist-led diabetes management services should be encouraged to ensure harmonization in the service, and making it possible for more research to systematically explore the effectiveness of individual or combination of intervention strategies provided.

## Supplementary Information

**Additional file 1**.

## Data Availability

All data relevant to the study are included in the article or uploaded as supplementary information.
