# Supplementary material for: The intervention strategies and service model for pharmacist-led diabetes management: a scoping review
Source: BMC Health Serv Res. 2023 Jan 18;23:46. doi: 10.1186/s12913-022-08977-1 (PMC9847048; doi:10.1186/s12913-022-08977-1)
Supplement: Supplementary file 1 — Additional file 1. [file 12913_2022_8977_MOESM1_ESM.docx]

| **Database** | **Date assessed** | **Search string** | **Results** |
| --- | --- | --- | --- |
| Pubmed | 5 November 2020 | ((diabetes OR "type 2" OR "diabetes mellitus") AND (pharmacist*) AND (medication AND adherence) OR (medication AND review) OR compliance OR concordance OR adherence OR education* OR intervention OR (medication AND therapy) OR (clinical AND review) OR nutrition OR diet* OR behaviour OR psych*)) | 2449 |
| SCOPUS | 5 November 2020 | TITLE-ABS-KEY((diabetes OR "type 2" OR "diabetes mellitus") AND (pharmacist*) AND (medication AND adherence) OR (medication AND review) OR compliance OR concordance OR adherence OR education* OR intervention OR (medication AND therapy) OR (clinical AND review) OR nutrition OR diet* OR behaviour OR psych*)) | 857 |
| Web of Science | 5 November 2020 | TS((diabetes OR "type 2" OR "diabetes mellitus") AND (pharmacist*) AND (medication AND adherence) OR (medication AND review) OR compliance OR concordance OR adherence OR education* OR intervention OR (medication AND therapy) OR (clinical AND review) OR nutrition OR diet* OR behaviour OR psych*)) | 717 |
| OVID | 5 November 2020 | ((diabetes OR "type 2" OR "diabetes mellitus") AND (pharmacist*) AND (medication AND adherence) OR (medication AND review) OR compliance OR concordance OR adherence OR education* OR intervention OR (medication AND therapy) OR (clinical AND review) OR nutrition OR diet* OR behaviour OR psych*)).ab,kf,ti | 347 |
